# Healthcare retention and clinical outcomes among adolescents living with HIV after transition from pediatric to adult care: a systematic review

**DOI:** 10.1186/s12889-020-09312-1

**Published:** 2020-08-03

**Authors:** Tiarney D. Ritchwood, Vincenzo Malo, Cameron Jones, Isha W. Metzger, Millicent Atujuna, Rebecca Marcus, Donaldson F. Conserve, Lara Handler, Linda-Gail Bekker

**Affiliations:** 1 Department of Family Medicine and Community Health, Duke University, 2200 W Main St, Durham, NC 27705 USA; 2 Duke Global Health Institute, Duke University, Durham, NC USA; 3 Department of Public Health Sciences, School of Medicine, Medical University of South Carolina, Charleston, SC USA; 4 Department of Psychology, University of Georgia, Athens, GA USA; 5 Desmond Tutu HIV Centre, Health Sciences Faculty, University of Cape Town, Institute of Infectious Disease, Cape Town, South Africa; 6 Department of Health Promotion, Education, and Behavior, Arnold School of Public Health, University of South Carolina, Columbia, SC USA; 7 School of Information and Library Science, University of North Carolina, Chapel Hill, NC USA

**Keywords:** Healthcare transition, HIV, Pediatric, Adolescent, Adult, Outcomes

## Abstract

**Background:**

Adolescents living with HIV (ALWH) who transition from pediatric to adult care face several challenges that increase their risk of experiencing treatment interruptions and being lost to HIV care with resultant increased morbidity and mortality. To date, few studies have examined their outcomes post-healthcare transition (HCT), precluding the development and dissemination of evidence-based interventions aimed at retaining ALWH in HIV care both during and after HCT. We conducted a systematic review to synthesize the outcomes of ALWH post-HCT to provide suggestions for future directions.

**Methods:**

We systematically searched several electronic databases through October 2019 using keywords for HIV, HCT and ALWH. We categorized studies by target population, country (i.e., upper-high income and low-middle income), study design (i.e., descriptive, mixed methods, quantitative), outcomes measured, and follow-up period.

**Results:**

A total of 24 studies met inclusion criteria. Studies were categorized according to the following HCT outcomes: retention in HIV care post-HCT (*n* = 13), changes in CD4+ count and viral load post-HCT (*n* = 16), and mortality among ALWH post-HCT (*n* = 7). Most studies (*n* = 11) examining retention in HIV care indicated that more than 70% of ALWH were retained in care 1–2 years post-HCT while the remaining studies (*n* = 2) reported retention rates less than 55%. While studies indicated that CD4+ counts and viral loads tended to worsen during the first few years post-HCT, these differences were often not statistically significant. Among all ALWH who transitioned to adult care, a small proportion died within their first seven years post-HCT. Among qualitative studies, common themes included transition readiness (*n* = 6), provider-patient relationship in the adult clinic setting (n = 6), and concern about the adult clinic setting (*n* = 4).

**Conclusions:**

Transition outcomes were poorest for ALWH with unsuppressed viremia pre-HCT, suggesting that this subgroup of ALWH may need greater support from their treatment teams and caregivers during and post-HCT to improve clinical outcomes.

## Background

The burden of HIV among adolescents between 10 and 19 years of age has been the focus of much attention and resources within the past decade [1]. Of the 1.8 million adolescents living with the HIV (ALWH) between the ages of 10 and 19 years [2], most ALWH were vertically infected due to maternal-to-child transmission (approximately 70%) [3]. Nearly 71% of all ALWH reside in one of 10 countries, with nine of these countries being in sub-Saharan Africa [4]. Adolescent girls accounted for around 60 to 74% of all ALWH between the ages of 15 and 19 years [4–6], with nearly 65% of infections being attributed to heterosexual sex, which is disproportionately higher than that of their male peers, in which 43% were due to horizontal infection [4].

Over the past three decades, the global scale up of antiretroviral treatment (ART) programs has made it possible for ALWH to live longer and healthier lives [2]. However, ALWH face substantial challenges with living healthily, which requires that they attend clinic appointments with their HIV care providers (i.e., be retained care) and adhere to strict ART regimens [7]. Of all age groups, ALWH have the lowest rates of retention in HIV care and ART adherence [8]. Globally, in 2018, only 54% of children living with HIV (ages 0–14) were retained in HIV care and only 41% were virally suppressed [9], which falls well below 90–90-90 treatment targets, which includes calls for 90% of all people living with HIV to adhere to ART and 90% to be virally suppressed [2]. Suboptimal rates of treatment retention and adherence among ALWH is directly linked to higher rates of HIV-related morbidity and mortality within this population, as AIDS-related illnesses are among the top 5 leading causes of deaths among adolescents, annually [10].

While there are a number of socio-structural determinants associated with poor health outcomes among ALWH [11–14], previous research has identified the transition from pediatric to adult care, frequently referred to as healthcare transition (HCT), as a particularly vulnerable period that influences health outcomes among ALWH [15–21]. HCT coincides with a time period during which many ALWH experience treatment interruptions and are at increased risk of loss-to-follow-up care (LTFU) only to return when critically ill [15]. In resource-limited settings, HCT often occurs at age 15 or younger, while in high-resource settings HCT occurs between ages 17 and 24 [16]. As such, differences in the timing and regional setting in which HCT occurs may have a significant impact on ALWH’s outcomes [17].

HCT should ideally represent a planned and dynamic process during which a team of healthcare professionals meets the psychological, biological and social needs of adolescents living with chronic conditions to prepare them to transition from pediatric to adult care [16, 22] However, in daily clinical practice, this process may be less structured. When HCT is structured and well-coordinated, ALWH have the opportunity to develop key skills that could enable to them effectively engage in self-management activities [23]. ALWH, for example, would presumably receive critical assistance from trained providers and caregivers to gradually gain independence in healthcare management responsibilities, ultimately enabling them to succeed in adult treatment settings [24]. However, when HCT is unstructured and poorly coordinated, ALWH may become disengaged from HIV care [20], resulting in early morbidity and mortality, and risk of forward transmission [25].

Despite the plethora of published guidelines and transition protocols, there is little uniformity with regard to the way in which pediatric HIV clinics approach HCT, making it difficult to identify best practices and develop efficacious interventions [16–21]. To date, there is little research aimed at determining whether there are significant differences in biologic measures (i.e., healthcare retention, treatment adherence, and survival over time) after ALWH transition to adult care and whether participation in HCT interventions improves outcomes among ALWH [21]. Therefore, the purpose of this systematic review is to synthesize research on the clinical outcomes of ALWH post-HCT, identify current gaps in the literature, and provide suggestions for future directions based upon observed findings.

## Methods

### Inclusion criteria

We included articles if they: (a) focused on ALWH aged 10–21 years who had completed HCT; (b) included data concerning what happens to ALWH post-HCT (i.e., retention in HIV care, mortality (i.e., death after HCT), and/or biologic measures approximating ART adherence); (c) presented pre- and post-HCT data for relevant outcomes; (d) were written in English; and (e) were published in peer-reviewed journals. We included children as young as 10 years of age because a number of guidelines recommend that children begin transition-related activities early and prior to actual transition, which may occur for adolescents as young as 13 years of age. Moreover, we included studies reporting mean adolescent age up to age 21 due to the fact that many ALWH, particularly those in high-resource settings, still receive pediatric HIV care services and have yet to transition to adult care settings.

#### Types of studies

Due to our interest in categorizing the clinical outcomes of ALWH post-HCT, this review focuses on quantitative and mixed methods studies. Quantitative studies include randomized or non-randomized controlled trials, retrospective or prospective cohort studies, case control studies, analytic cross-sectional studies and descriptive epidemiological studies. Mixed methods studies include those that provide descriptive data on ALWHs’ outcomes post-HCT.

### Search strategy

The details of our search strategy have been previously recorded [26]. In summary, we followed Lipsey and Wilson’s (2001) suggestions on conducting systematic reviews [27, 28]. This review also complies with the Preferred Reporting Items for Systematic Reviews and Meta-analyses (PRISMA) guidelines (see the PRISMA Checklist in the supplementary files). Specifically, we began by searching the following electronic databases in October 2019 due to their interdisciplinary focus: PubMed, CINAHL, Web of Science, Global Health, and Embase. We used the following search terms (where “*” indicates the use of a wild card) to identify adolescents: child* OR pediatric* OR adolescen* OR youth* OR young adult*. To narrow our search to articles focused on HCT, we used the following terms: (continuity OR transition*) OR continuity of patient care AND (services OR care) OR transition to adult*. We also reviewed the bibliographies of studies meeting inclusion criteria to identify other potential studies to include in our review. Two members of the research team completed three rounds of screening: the title, abstract, and full-text screenings. All discrepancies were resolved through consensus.

### Data extraction and analysis

For each article, we documented the following information: country (i.e., high-income, low-middle income, mixed income), setting, description of pre- and post-transition process, study design (i.e., descriptive, mixed methods, quantitative), sample size, participant demographics, follow-up length, and the HCT outcomes reported. The study team resolve discrepancies through group discussion. One member of the study team conducted the study quality assessment using the Critical Appraisal Skills Programme (CASP) tool [29]. The maximum score on the CASP for quantitative studies is 17 points and 10 points for qualitative studies. A second member of the research team reassessed scores of less than 10 points for quantitative and less than 7 points for qualitative studies. All discrepancies were resolved via group discussion and consensus. Scores for each included manuscript are available in the supplementary file linked to this manuscript (see Additional File 1).

## Results

### Search results

**Fig. 1:**
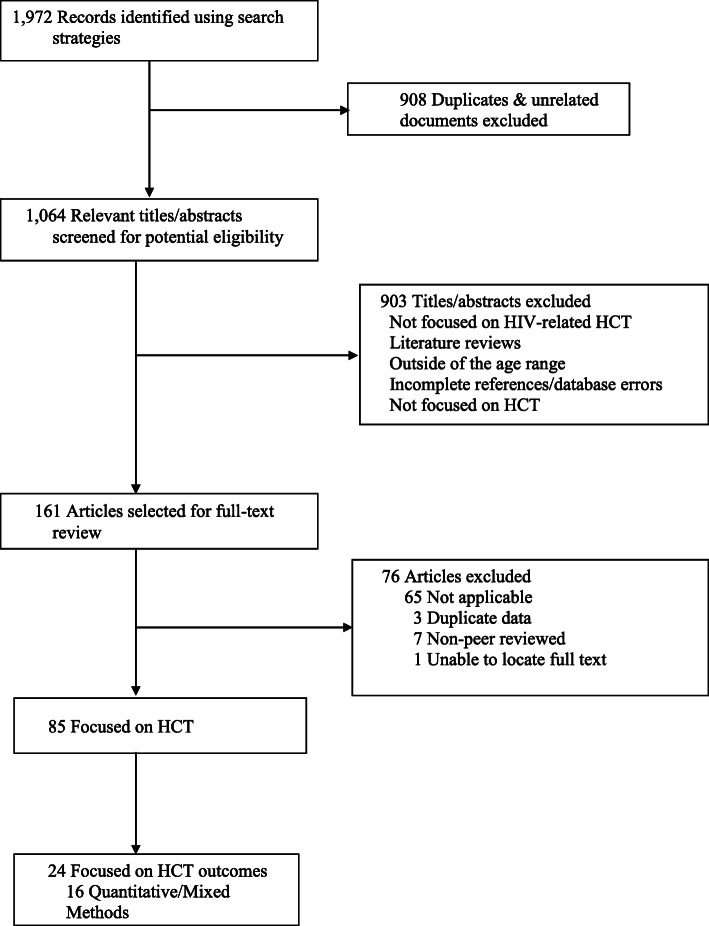
Flow diagram of literature reviewed for a systematic review of the treatment outcomes among adolescents transitioning from pediatric to adult care. A total of 1972 relevant titles were assessed. After removing duplicates and unrelated titles and abstracts, 1064 relevant manuscripts were identified. Of these only 85 studies focused on healthcare transition and 24 focused on HCT outcomes among adolescents living with HIV

As illustrated in Fig. 1, the initial search generated 1972 titles/abstracts. After removing duplicates and excluding studies that did not meet inclusion criteria, 175 studies remained. Of these, 69 articles focused on HCT for ALWH. Further refinement based on inclusion of pre- and post-HCT data for quantitative studies and transition outcomes for qualitative studies resulted in a sample of 16 quantitative [30–45] and 8 qualitative studies [12, 46–52].

### Study quality assessment

Most studies in this review used longitudinal or pre/post measurements, with several reporting the transition results of ALWH who had participated in a formal HCT program. None of the studies included a comparison group or random selection. While most studies in this review scored well on quality, there were no randomized controlled trials, limiting our ability to evaluate the efficacy of the HCT interventions described. Moreover, included studies tended to have small samples ranging between 24 and 72 participants. The average quality score was 12.44 for quantitative articles, indicating that most studies in this area ranged between low to moderate quality, while qualitative articles had an average score of 9.3, indicating most were of good quality.

### Description of included studies

A total of 24 studies are included in this review. Study designs varied between quantitative, including descriptive and mixed-methods studies (see Table 1), and qualitative studies (see Table 2). The quantitative/mixed-methods studies reviewed reported on three main outcomes: 1) whether the adolescent was retained in care post-HCT (*n* = 13); 2) whether the clinical measurements of CD4 count and viral load (VL) changed between pre- to post-HCT (*n* = 16); and 3) rates of mortality post-HCT (*n* = 7). Of the 16 quantitative studies, 15 reported two or more of these outcomes with 5 (33.3%) reporting all three. Most of the studies focused on youth from high-income countries, including the United States (*n* = 8), United Kingdom (*n* = 4), Italy (n = 1), Canada (n = 1), Poland (n = 1), Sweden (n = 1), Netherlands (n = 1), and France (n = 1). Only six focused on youth from low-middle income countries: Brazil (n = 1), Thailand (n = 1), Uganda (*n* = 2), and South Africa (n = 2). Most participants were between 17 and 22 years of age, female, and of African descent. Settings ranged from HIV specialty clinics, university medical centers, public hospitals, and medical research facilities.

**Table 1:** Study description table

Authors	Location/Design	Population	Retention in HIV Care	Viral Load (VL)/CD4+ count	Mortality
Davies et al., 2017 [34]	• Cape Town, South Africa • Longitudinal • PHIV • Setting: two hospitals and two primary care clinics	• M_age_ at transition = 10–19 years • *n* = 460 • Sex = 53% female • Duration of follow up = 3.3 years	Evaluated longitudinal outcomes of ALWH post-HCT and found that 81% had a successful transition. One year later, retention was 90% but declined to 84% at 3 years. Retention was lower in adolescents 15–19-year compared with those who were 10–14-years of age up to 2 years post-HCT although at 3 years figures for both groups were similar.	78% percent of patients were virologically suppressed during transition 64% of these patients had CD4 > 500 cells/μL.	None reported
Fish et al., 2014 [39]	• UK & Ireland • Longitudinal • PHIV • Setting: outpatient clinics, data from network of health professionals	• Median age at HCT for 11 deaths = 17 years • Median age at death after HCT = 21 years • Race/ethnicity: 82% Black African; 18% White European • *n* = 248 • Sex = not reported	Not applicable	At death, the median CD4 count was 27 cells/μL. While 5 patients were on ART, only 2 had a VL < 50 HIV-1 RNA copies/mL.	Conducted a multicenter audit to assess the number of deaths and associated factors among PHIV after HCT. A total of 11 participants died during assessment period of 5 years. Causes included suicide (n = 2), AIDS (*n* = 7) and bronchiectasis (*n* = 1). Cause of death for one patient was unknown. 82% had poor adherence beginning in pediatric care, were ART resistant, and had comorbid mental health diagnoses.
Griffith et al., 2019 [40]	• United States • Retrospective cohort • PHIV and nPHIV with at least one visit in adult clinic after HCT • Setting: 2 urban HIV care programs	• *N* = 89 • Sex = 62% female • Race = 81% African American • 75% on ARTs before HCT	Evaluated retention in care, CD4 count and VL post-HCT. A total of 79 (89%) patients were successfully retained in care one year after transition, of which 53 had stable or improved viral loads.	57% of participants had viral loads less than 400 copies/mL pre-transition. 51% of patients were virally suppressed post-HCT.	None reported
Haghighat et al., 2019 [35]	• Eastern Cape province, South Africa • Longitudinal • Prospective cohort • Setting: health care facilities	*• N* = 951 • Sex = 54.3% female • Median age at study enrollment = 13 years • 26.1% horizontally infected • Median age at ART initiatio*n* = 9 years	Characterized clinical outcomes and mobility through VL, mortality, and LTFU. 550 persons (57.8%) started ART in a pediatric setting, of which 35.3% transitioned to adult care. Median age at transition was 14 years old. Of the 35.3% to transition, 91.2% were retained in care 18 months later. 84 ALWH (8.8%) were LTFU, defined as missing all appointments within the past 3 months and being untraceable.	Of the 143 ALWH to transition to adult care who had pre and post HCT VL data, there was no change in .	Out of those who transitioned to adult care 7 died (3.6%).
Hansudewechakul et al., 2015 [37]	• Chiang Rai province, Thailand • Longitudinal • PHIV • Setting: public hospital	• Age = not reported • Race/ethnicity: 100% Thai • *n* = 67 • Sex = not reported • 1–5 years post-HCT	Evaluated the outcomes of PHIV after completion of a voluntary HCT camp intended to prepare PHIV for adult care. PHIV were transitioned to adult care in groups rather than individually to facilitate adherence modeling and social support. A total of 73% of ALWH were retained in care 2 to 5 years post-HCT depending on when they were enrolled., while13% were LTFU.	Pre-HCT biologic data were not reported. Post-HCT, 37 had a VL < 40. The remaining youth had VLs between 40 and 1999 (n = 6) and > 2000 (n = 9).	Four participants died post-HCT.
Hussen et al., 2017 [41]	• Atlanta, Georgia, United States • Retrospective cohort study • PHIV & BHIV • Setting: HIV clinic, public hospital	• Median age at last pediatric visit = 23.8 years (22.0–24.8 years) • *n* = 72 (last pediatric visit) • Sex = 62. 5% male • Race/ethnicity: 93% Black African; 3% Mixed; 2% White	Evaluated retention in HIV pre-, during and post-HCT. Of 72 ALWH, 89% were retained in HIV care one year post-HCT and 56% were in care 2 years post-HCT. ALWH with suppressed viremia during the last pediatric visit were more likely to be suppressed post-HCT.	Of all transitioned ALWH, 49 had suppressed viremia one year post-HCT, or 53% of ALWH.	None reported
Izzo et al., 2018 [31]	• Brescia, Northern Italy. • Retrospective • PHIV • Setting: outpatient clinic	• M_age_ at HCT =18 years • *n* = 24 • Sex = 37.5% male • 75% were Italian.	None reported	Described the viro-immunology outcome of PHIV. Pre-HCT, the median CD4+ T-cell count was 534 cell/lL, 62.5% had HIV-RNA < 50 copies/mL and 25% had HIV-RNA 50–10,000 copies/mL, and 12.5% had HIV-RNA > 10, 000 copies/mL. Post-HCT, 5 patients were LTFU (median 52 months), median CD4+ T-cell count was 716 cell/lL, 100% had HIV-RNA < 50 copies/mL, 0% had HIV-RNA 50–10,000 copies/mL and HIV-RNA > 10, 000 copies/mL.	None reported
Judd et al., 2017 [38]	• United Kingdom • Longitudinal • PHIV • Setting: clinic settings, cohort data	• Median age pre-HCT = 17 years (16–18 years) • Race/ethnicity: 80% Black African; 11% White; 9% other • *n* = 271 • Sex = 53% female • 15.4 years duration of total follow up	Not reported	Median CD4+ count at 12 months pre-HCT was 465 cells/mm^3^ and post-HCT was 460 cells/mm^3^. Pre-HCT, 21% had CD4 < 200 cells/mm3 at least once in the 12-month periods, post-HCT 23% had CD4 < 200 cells/mm3 at least once in the 12-month periods. For those on ART pre-HCT 28% had two consecutive VLs (≤6 months apart) > 400 copies/mL or one VL > 10,000 copies/mL in the 12 month periods, post-HCT 29% had two consecutive VLs (≤6 months apart) > 400 copies/mL or one VL > 10,000 copies/mL in the 12 month. Pre- HCT, 47% had two consecutive VLs > 400 copies/mL or one VL > 10,000 copies/mL in the 12-month periods regardless of ART status which was 52% post-HCT.	Clinic records indicated that 7 participants died in adult care. Causes of death were AIDS (*n* = 3), renal failure (*n* = 1), leukoencephalopathy (n = 1), pulmonary tuberculosis (n = 1). The cause of death was unknown for one youth. 71% had periods when they were not receiving ART prior to death.
Kakkar et al., 2016 [30]	• Québec, Canada • Descriptive • PHIV • Setting: HIV clinic	• M_age_ = 22 (19–25 years) *n* = 54 • Sex = 40% male • *n* = 45 • Race/ethnicity: not reported • M = 3.6 years post HCT (range 1.1–6.8 years)	The researchers reviewed the clinic records of PHIV who had transitioned to adult care. Of the 25 who agreed to study participation, 76% were retained in HIV care at follow-up of one year post-HCT or later and eight were LTFU.	Pre-HCT, 64% had a CD4 count > 500 cells/mm^3^, 16% between 200 and 500 cells/mm^3^, and 20% were immunosuppressed with a CD4 count < 200 cells/mm^3^. Moreover, fewer PHIV had VLs greater than 500 cells/mm^3^ (decreased from 64 to 29%). When PHIV were asked how often they missed drug doses in the past month, 40% reported no missed doses, 28% reported occasional missed doses, 16% reported frequently missed doses, and 12% had stopped all ARV therapy. Of the 16 PHIV for which VL data were available, 9 remained undetectable, one had an increase in VL to a detectable status, and 6 remained detectable.	Of the 45 who transitioned, 4 were deceased.
Kowalska et al., 2019 [33]	• Warsaw, Poland • Cross-sectional • Setting: pediatric health care	• *N* = 30/120 transferred to adult care • Median age at transfer = 19.1 years • Median age at diagnosis = 53 months • Median post-HCT follow-up = 1.9 (0.7–4.5) years	Most ALWH (83%) were retained in care at a median follow up time of 1.9 years post-HCT. Four patients were LTFU, three who were not virally suppressed in pediatric care and one who was virally suppressed in pediatric care.	Twenty-one percent were virologically suppressed pre-HCT. Of these, seventeen maintained suppressed viremia post-HCT. Among ALWH who were not virologically suppressed pre-HCT (*n* = 9), three achieved suppression in adult care.	One out of the 9 patients who were virologically non-supressed in pediatric care died post-HCT from drug overdose.
Maturo et al., 2015 [42]	• Miami, Florida, United States • Longitudinal & Descriptive • BHIV & PHIV • Setting: HIV clinic, university hospital	• M_age_ at HCT follow up = 17.55 (18–29 years) • Race/ethnicity: 84% African American; 13% Hispanic; 3% Mixed race • *n* = 38 • Sex = 44.8% males, 2.6% transgender • Did not report years post-HCT	Data are from a HCT program for PHIV (11%) and BHIV (89%) that consisted of five distinct phases. The authors reviewed the medical records of individuals who participated in the program and results indicated that 47% completed the program and were retained in adult care 1 year post-HCT. Among those who did not complete the program, 8% were still in the program.	Pre- HCT, ALWHs’ mean CD4+ count was 479 cells/mm^3^ (range = 4–1255) and their mean HIV RNA level was 18,528 copies/mL (range: 130–114,800). About 26% reported adherence issues. Post-HCT, 22% reported adherence issues. Their mean CD4+ count was 604 cells/mm^3^ (range = 185–1124) and their mean HIV RNA level was 11,488 copies/mL. Follow-up data were only available for seven of the non-completers and indicated that 29% reported adherence issues. Their mean CD4+ count was 603 cells/mm^3^ (range = 4–1, 1255) and their mean HIV RNA level was 15,294 copies/mL (range: 395–66,683).	None reported
Tassiopoulos et al., 2019 [43]	• United States • Cohort study • PHIV	• *N* = 455 • Median age = 21.5 years • Sex = 61% female • Race = 68% Black	124 participants transitioned to adult care. Mean age at last pediatric visit was 21.7 years. 59% of those who transitioned missed an ARV dose in the past 3 months, as compared to 60% who did not transition.	Median CD4 count was 402 cells/mm^3^ for those who transitioned compared to 535 cells/mm^3^ for all 455 patients prior. Of the 124 who transitioned, 56% had at least one instance of unsuppressed VL prior to transition compared to 31% with an unsuppressed VL within a year post transition	None reported
Westling et al., 2016 [36]	• Stockholm, Sweden • Cross-sectional & Longitudinal VPHIV & BHIV • Setting: transition clinic, university hospital	• M_age_ = 19 (17–25 years) • *n* = 34 • Sex = 50% male • 2 years post-HCT	Described a HCT program in which PHIV/BHIV met with physicians, nurses and counselors from a pediatric clinic for 1–2 years. Twenty-nine reported at the 2-year follow-up, of which 23 underwent HCT.	Prior to HCT, 88% were on ART for a median of 9 years, 6% experienced treatment interruption, and 75% had a history of adherence issues. Participants experienced mutations against at least two drug classes (25%), treatment resistance requiring changes to medication (40%), only 14% had a CD4 cell count that was less than < 350 × 106/L, and 90% (27 of 30) of those on ART had a VL of < 50 copies/mL. At the 2-year follow-up, 90% (26 of 29) of those on ART had a VL of < 50 copies/mL, 92% had a VL < 50 copies/mL, and 19% experienced treatment interruptions.	None reported
Weijsenfeld et al., 2016 [32]	• Amsterdam, Netherlands • Longitudinal • PHIV & BHIV • Setting: university medical centers	• M_age_ at diagnosis = 8 (3–13 years); M_age_ at transition = 19 (18–20 years) • *n* = 58 • Sex = 42% male • 1–6 years post-HCT	Evaluated virological and social outcomes of PHIV/BHIV prior to and after HCT, and identified factors associated with VF. Results indicated that 86% were retained in HIV care and 14% were LTFU at a mean follow-up time of 1.5 years.	Youth reporting low (OR, 3.32 [95% CI, 1.39–7.92], *P* = .007) or unknown (OR, 6.23 [95% CI, 2.04–18.9]) educational attainment were more likely than their peers with higher levels of attainment to experience VF. Youth who lacked autonomy in treatment adherence at transition were more likely than their peers with autonomy to experience VF (OR, 6.89 [95% CI, 2.57–18.5], *P* < .001). Approximately 50% of all ALWH in this study experienced VF in pediatric care and of these, 20 (34%) also experienced VF in adult care. For those without VF experiences in pediatric care, only 2 (3%) experienced VF as adults.	None reported
Wiener et al., 2011 [44]	• United States • Mixed methods: pre/post HCT • PHIV & BHIV • Setting: medical research facility	• M_age_ = 22 (18–31 years) • *n* = 59 • Sex = 51% male • 4 years post clinic closure	Described ALWH (18+ years) participating in a pediatric HIV care program. Data were gathered between September and December 2008, which is the time when the program was terminated. Follow-up occurred in 2008. Results indicated that 55 ALWH (93%) had been retained in HIV care, of which 42 (71%) transitioned to adult care. Of those who experienced HCT, 45% reported that the transition was more difficult than expected; 86% were on ART; and 45% reported difficulties adhering to treatment.	There appeared to be a trend towards lower CD4 counts for those who experienced HCT with pre-HCT mean of 575 and post-HCT mean of 504. The difference in change was not significant.	
Xia et al., 2018 [45]	• New York City, United States • Retrospective pre-post study • Matched exposed/unexposed nested cohort study • PHIV • Setting: NYC HIV surveillance registry	• *N* = 1791 • Sex = 52.7% female • Mean age of transition = 22 years old	Reported transition duration, retention in care, and pre-post CD4 cell count. A total of 735 ALWH transitioned to adult care. Of those for which 3-year follow-up data were available after the first adult visit, 367 ALWH, (94.7%) remained in care.	One year post-HCT, 337/694 (48.6%) were virally suppressed. In year 2, 301/589 (51.1%) were suppressed, and in year 3247/477 (51.8%) were suppressed.	41 persons died in the first year of transition. 82.9% were from HIV-related diseases.

Note: *PHIV* adolescents with perinatal HIV infection, *BHIV* adolescents with behavioral HIV infection, which includes transfusion, IV drug use, and sexual routes of transmission, *HCT* healthcare transition, *VF* virologic failure, *LTFU* lost to follow-up care

**Table 2:** Qualitative studies

Authors	Location/Design	Population	Themes
Hussen et al., 2019 [46]	• United States • Qualitative semi-structured focus groups	• N = 24 • Providers in four groups (2 from pediatric clinic, 2 from adult clinic)	Three types of HCTs were described: • Ideal transitions would include HCT planning a year in advance and prepare ALWH for a major shift. • Abrupt transitions that result from medical needs are linked to treatment non-adherence • De Facto transitions where adolescents disengage in care and then re-engage in care after turning 25 years old Poor engagement in pediatric care was linked to poor engagement in adult care.
Bundock et al., 2011 [12]	• Australia, UK • Cross-sectional study comparing patient satisfaction at a U.K. HIV transition clinic and an Australian diabetes transition clinic	• *N* = 21 PHIV in UK • Sex = 57% female • *N* = 39 young people with diabetes in Australia • Sex = 56% female	• 18/19 PHIV reported an easy transition process compared to 34/39 of those with diabetes who felt their transition to adult care was easy. • 13/19 PHIV and 28/39 diabetes patients reported their HCT had a positive impact on their health. • All PHIV reported the transition clinic to be preferable to the adult clinic • The most important concerns were the staff’s ability to communicate with young people, preparation for the physical transition and transition in responsibility, and feeling comfortable discussing personal health.
Katusiime et al., 2013 [47]	• Uganda • Thematic analysis of semi-structured interviews with adolescents post-transition	• *N* = 30 • Patients at least 1 year after HCT	The study identified six major themes: • Adjustment to health care providers • Adult clinic logistics • Positive attributes of adult clinic, like specialized care • Transfer to other health centers • Perceived sense of stigma • Patient recommendations for staff in adult clinic such as gaining experience working in transition or pediatric clinic
Machado et al., 2016 [48]	• Brazil • Thematic analysis of semi-structured interviews with adolescents	• N = 16 ALWH who were part of transition protocol • Sex = 50% female • Median age = 17 years	• Participants noted turning points in their lives related to their transition and identified social support as a pivotal factor to dealing with it. • The bond between pediatric providers and patients was another important aspect of care, so HCTs brought concerns about disruption and abandonment. • Adult care was negatively perceived. • ALWH recommended more time to adapt during the HCT process and more communication between providers in both settings.
Miles et al., 2004 [49]	• UK • Thematic analysis of semi-structured interviews with adolescents	• N = 7 • Median age = 16 years	Themes were identified based on transition phase (pre, during, post) with subthemes identified for each. • Pre-transition: Participants identified the benefit of introductions to adult-care providers and anxieties about care coordination and the adult environment • The actual transition: Four participants found the transition easy, while three delayed their transition due to greater trust in pediatric clinic • Post-transition: All participants identified benefits of transition, most identified importance of losing relationships with pediatric clinic physicians, and recommendations were made about pre-transition visits and youth-friendly environments.
Sharma et al., 2014 [50]	• USA • Grounded theory analysis of structured interviews with adolescents and their guardians	• *N* = 15 youths, 8 caregivers • Mean age of patients = 18 years	Three major themes were identified: • Lack of preparation for the HCT expressed by both youth and caregivers • Anxiety about changing providers and health care settings • Concerns about increase in responsibility with time
Valenzuela et al. 2011 [51]	• USA • Thematic analysis of semi-structured interviews with adolescents	• N = 10 patients who completed HCT • Mean age = 26.7 years	Six themes were identified: • Providers acting as family in adolescent care • Adolescent care as a time to learn about the disease and grow • Anxiety and lack of preparation for HCT Recommendations for improving HCT • Change in experience of care with shift to adult care • Opportunities for growth in adult care
Le Roux et al., 2017 [52, 53]	• France • Thematic analysis of semi-structured interviews with health providers	• *N* = 18	Three major problems during transition that were identified included: trouble accepting the disease, communication challenges in linking from pediatric to adult care, and difficulty navigating the new health care environment.

### Adolescent retention in HIV care post-HCT

Of the 16 quantitative studies, 13 reported data regarding retention in care post-HCT. Most of the studies were from high-income countries in North America, followed by Europe and low-middle income countries, respectively. We found that retention rates across studies ranged from 37 to 94.7%, with the majority of studies reporting that most ALWH who underwent HCT were retained in treatment at least 1–2 years later. However, as the follow-up periods grew in length, the rates of retention in HIV care declined. Factors that appeared to negatively influence treatment retention post-HCT included older age, having a detectable VL, or instability at home, as indicated by involvement in the Child Protective Service [41, 42]. In a Canadian study, for example, of the 45 ALWH who transitioned to adult care, 76% remained engaged in care (defined as having at least one visit with a clinician in the previous 6 months) up to seven years post-HCT, with 12% reporting engagement in the past 12 months and 12% reporting that their last physician’s appointment was over a year ago [30]. An Italian study of 24 ALWH who transitioned to adult care found that 100% of youth were retained in care a year later, and 80% of these were retained in care 4 years post-HCT [31]. Of those who were LTFU (*n* = 5), 60% had detectable VLs (i.e., VL > 10,000 copies/mL) at both transition and last recorded visit. In a study of 58 Dutch ALWH, 86% had been retained in HIV care post-HCT, with a mean follow-up period of 1.5 years [32]. In this study, involvement with Child Protective Service predicted poor treatment retention (*p* = .02).

A South African study found that of 460 ALWH who transitioned to adult care, 81% had completed at least one clinic visit post-HCT [34]. One year later, 90% of these were retained in care and 84% of these were still in care 3 years later. The researchers found that retention was lower in 15 to 19-year-olds when compared to 10 to 14-year-olds up to two years post-HCT, though retention rates were similar three years post-HCT. A study of 951 ALWH in South Africa, 64.7% chose to remain in pediatric care rather than transition to adult care [35]. Of the 35.3% who transitioned, 91.2% were retained in care 18 months later, with the remainder being LTFU, which was defined as missing all scheduled appointments within the past 3 months and being untraceable.

### Change in CD4+ count and VL post-HCT

Sixteen studies reported data on change in CD4+ count and VL post-HCT. Most of the studies were from high-income countries in North America, followed by Europe, followed by two from South Africa. Overall, our findings suggest that CD4+ counts and VLs were either stable or showed slight improvements post-HCT within the first few years. However, as with retention in HIV care, these biologic measures of treatment adherence tended to worsen over time. While few studies investigated the impact of age on these measures, it appears that those between 18 and 19 may be particularly vulnerable to virologic failure [32].

In a US-based study, median CD4 count was 402 cells/mm^3^ for those who had transitioned to adult care (*n* = 124) compared to 561 cells/mm^3^ [*p* = .01] in those who had not transitioned (*n* = 331) [43]. More than half of ALWH who transitioned to adult care had at least one test that indicated unsuppressed viremia within the year before their HCT. African American participants were at a greater likelihood to have unsuppressed viremia than other ethnic groups [*p* = .04]. Another study US-based study found that, of the 735 ALWH who had successfully transitioned to adult care, in addition to trends indicating poorer retention in HIV care over a three-year period, proportions of ALWH with suppressed viremia were suboptimal, ranging from approximately 49–52% [45], which are similar to estimates reported in other studies [40, 45]. Data from a Canadian cohort of 54 youth indicated that pre-HCT, most ALWH (64%) had a CD4+ count > 500 cells/mm^3^, 20% had a CD4+ count of <200cells/mm^3^, and 16% had a CD4+ count between 200 and 500 cells/mm^3^ [30]. One year post-HCT, 41% of ALWH had a CD4+ count between 200 and 500 cells/mm^3^, followed by those with counts that were > 500 cells/mm^3^ and those with counts that were < 200cells/mm^3^ (both 29%). Sixty percent had an undetectable VL prior to HCT, with all others having greater than 1000 copies/mL. A year later, VLs for only 16 patients were available, including 10 who had undetectable VLs pre-HCT. Of the 10 patients with undetectable VLs, 9 remained undetectable and one experienced an increase in VL from fewer than 40 copies/ml to 1759 copies/ml.

In a Dutch HCT study of 59 ALWH, 48% of whom were born in sub-Saharan Africa, a total of 50% experienced virologic failure (i.e., 2 consecutive VLs > 400 copies/mL) pre-HCT, of whom 34% also experienced virologic failure post-HCT [32]. Moreover, ALWH between 18 and 19 years of age were more likely to experience virologic failure around the time of transition (odds ratio [OR], 4.26 [95% confidence interval (95% CI, 1.12–16.28], *p* = .03); had less autonomy over medication management (OR, 6.89 [95% CI, 2.57–18.5], *P* < .001); had less HIV knowledge (OR, 5.15 [95% CI, 2.16–12.3], P < .001); and lower educational attainment (OR, 3.32 [95% CI, 1.39–7.92], *P* = .007). A South African study of 460 ALWH found that, among those who were retained in care (84%), the proportion with VLs < 400 copies/ml and CD4+ > 500 cells/μl were similar up to two years post-HCT but declined 3 years post-HCT from 80 to 75% and 71 to 59%, respectively. Moreover, older adolescents (15–19 years) tended to have poorer viral control than their younger peers (10–14 years) [34].

### Mortality among adolescents post-HCT

Seven studies reported data regarding mortality among patients who transitioned from pediatric to adult care. Five studies were from high-income countries, and two were from low-middle income countries. Across studies, approximately fewer than 9% died post-HCT. ALWH who died post-HCT tended to have poor ART adherence and unsuppressed VLs at transition. Cause of death was linked to advanced HIV disease for most youth, though it is notable that some studies did not report the cause of death (*n* = 3). Moreover, some studies reported that the most ALWH who died post-HCT had at least one co-morbid non-communicable disease. However, few of the included studies investigated this relation.

An English study used data from 14 adult clinics to establish the number of transitioned patients who had died and identify factors associated with their deaths [39]. Of the 248 adults who were perinatally infected with HIV (PHIV), 4% died during the five-year assessment period. Records indicated that 81% of the deceased had persistently poor ART adherence both pre- and post-HCT, which was attributed to lack of treatment motivation, side effects, limited acceptance of HIV diagnosis, and poor adherence skills. Seven individuals died as a result of complications from advanced HIV disease, two committed suicide, and one had bronchiectasis. The cause of death could not be determined for one youth. All but one patient had comorbidities, such as pneumonia and psychiatric diagnoses (e.g., depression and eating disorder), at the time of their deaths. Lastly, the authors calculated the mortality rates of youth (13 years or older) who were PHIV and who received HIV care between 2006 and 2011 (*n* = 996). Results indicated that the mortality rate was higher in the older age groups. A study based in the UK found that, of 271 ALWH who transitioned to adult care, 3% died within 15.4 years [38]. The causes of death were as follows: complications due to AIDS (*n* = 3), leukoencephalopathy (*n* = 1), renal failure (n = 1), pulmonary tuberculosis (n = 1), and the cause of death for one participant was unknown. Similarly, data from 54 Canadian ALWH showed that 9% of ALWH had died within 7 years of transitioning to adult care [30]. One of the US studies reported 41 deaths within the first year of transition for the 735 patients who transitioned. 82.9% were from HIV-related diseases [45].

### Qualitative themes

Eight qualitative studies met inclusion criteria. We identified three cross-cutting themes that characterized findings regarding the outcomes of ALWH post-HCT: 1) transition readiness, 2) patient-provider relationship factors, and 3) concern about the adult clinic setting. These themes are described in detail below.

### Transition readiness

Half of all qualitative studies identified transition readiness as a key factor in successful post-HCT outcomes, which included being retained in HIV care and/or adhering to prescribed treatment regimens. While the HCT process varied by study, ALWH preferred a purposeful process in which they were able to meet the adult provider prior to HCT [51]. One U.S.-based study of pediatric and adult providers, for example, suggested that ALWH who participated in HCT processes that began at least a year prior to HCT and included a meeting between ALWH and adult provider, a tour of the adult clinic, and conversations about differences between the pediatric and adult clinics had better outcomes [46]. In a UK-based study of the ALWH who were transitioned from pediatric care to an adolescent HCT clinic, ALWH reported high levels of satisfaction with the adolescent setting, preferring it to the adult clinic due to its perceived youth-friendliness and developmentally-appropriate treatment of adolescent patients [12]. ALWH described the adult clinic as “scary” and did not feel ready to transition to adult care.

### Patient-provider relationship factors

Several studies identified the patient-provider relationship as an important contributor to successful HCT outcomes. ALWH described their pediatric providers as ‘family’, highlighting years of trust and rapport that have been established between the patient and provider [47, 51]}. In a Ugandan study, ALWH suggested that providers prepare ALWH for transition by helping them to understand that the adult providers will not provide the same “special attention” that they received from their pediatric providers [47]. In a Brazilian study, ALWH expressed concerns about severing their bonds with the pediatric care team, fearing abandonment and treatment disruption related to the time period required to adjust to a new provider and treatment setting [48].

### Concerns about the adult clinic setting

The majority of studies identified concerns about the adult clinic setting as a substantial barrier to successful HCT. ALWH expressed concerns about potential stigma in the adult clinic and interacting with staff who were not accustomed to working with youth [12]. A French study, for example, found that providers had challenges with understanding adolescent behavior and were unsure how best to interact with them [52]. Recommendations included training for adult providers to improve attitudes towards, and treatment of. ALWH [47]. Other concerns included anxiety related to navigating the adult treatment setting and the associated responsibility of managing their own care outside of the supportive pediatric environment [47, 51, 52].

## Discussion

The current review characterizes the outcomes of ALWH who transitioned from pediatric to adult HIV care and has identified three categories that describe the focus of previous quantitative findings regarding ALWHs’ transition outcomes: 1) retention in HIV care, 2) changes in CD4+ counts and VLs, and 3) mortality. It is notable that most of the included studies had a number of methodological weaknesses, including: lack of randomized controlled trials for intervention studies, small sample sizes, and non-standardized definitions of treatment retention and virologic failure [53]. We identified three overlapping themes from qualitative studies, including transition readiness, patient-provider relationship, and concerns about the adult clinic setting. The results of our review indicated that most ALWH had positive outcomes following transition to adult care, often being retained in HIV care years after transition, experiencing few deaths within the first few years after transition, and having stable VLs. While these findings may appear to be in contrast to what might be expected [54], we must note that the follow-up period for most studies was limited to 1–2 years post-HCT. Moreover, several included studies made use of retrospective cohort follow-ups rather than prospective measurements, limiting our ability to describe temporal associations among variables impacting ALWHs’ outcomes.

The proportion of ALWH who were retained in HIV care post-HCT varied by study but appeared to be highest within the first two years of being in adult care and tended to decrease over time. Moreover, some studies found that older ALWH had worse outcomes when compared to their younger peers [34, 41, 42]. Older ALWH are often given more responsibility for managing their HIV care upon HCT and along with challenges associated with adjusting to the adult clinic environment and navigating the adult system with less support than that provided in the pediatric setting may make it more difficult for older ALWH to remain in HIV within the first two years of adult treatment [7]. One study, however, showed that older ALWH had treatment retention rates that were similar to their younger peers between years two and three, indicating that longer follow-up periods may produce a different picture of HIV outcomes among older ALWH [34]. Most of the included studies reported transition outcomes for ALWH who had participated in a HCT program, which were usually characterized by at least one meeting between the pediatric and adult infectious diseases physician; coordination of services from an interdisciplinary team that included a mental health clinician; and intervention sessions focused on HIV education, treatment adherence, and/or disease self-management. However, there was a significant amount of variability in the types of services offered in transition programs. For example, programs varied in the degree to which providers from the adult clinics were involved in the transition process. Previous research from the United States, for example, found that staff and providers in adult clinics often played a passive role in HCT, attributing much of the responsibility for preparing ALWH for HCT to the pediatric or adolescent clinic [24]. This could be problematic, as several qualitative studies have suggested that negative perceptions of the adult treatment team or limited communication between the pediatric and adult HIV treatment teams serve as barriers to successful HCT [44, 48, 50, 51, 55, 56]. As such, active engagement of the adult treatment team during HCT may be critical to successful outcomes among ALWH. This could be facilitated through the implementation of inter-clinic protocols [24], in which the sending clinic (e.g., pediatric/adolescent) works collaboratively with the receiving clinic (e.g., adult) to develop clinic policies aimed at reducing HCT barriers. Such protocols could also mandate that adult treatment providers receive training in adolescent socio-cognitive development to support patient-centered care, which is consistent with previous research suggesting that training for both ALWH and adult providers facilitates successful HCT [57]. This level of collaboration may reduce reducing attrition rates and poor disease self-management practices post-HCT and is consistent with previous qualitative research suggesting that formal HCT protocols within clinics are associated with better outcomes among ALWH [18].

We found that most studies with analyses of post-HCT CD4+ counts and VL showed that biologic measures either improved or were stable post-HCT. While methodological variations preclude comparisons in CD4+ and VLs across studies, our results support calls for differentiated and specialized models of patient care for ALWH [58]. ALWH who have challenges with treatment adherence or who are not virally suppressed prior to HCT, for example, may require a greater level of attention post-HCT [31]. ALWH who were non-adherent to ART and/or had advanced disease progression pre-HCT may be more likely than their peers to be LTFU [31]. Inter-clinic protocols could assist the adult treatment team with developing developmentally-appropriate, patient-centered treatment plans to support these ALWH. Regarding studies assessing mortality post-HCT, we found that mortality rates up to 10 years post-HCT ranged between 4 and 9% of transitioned ALWH [30, 33, 35, 37–39, 45]. It is notable that the sample sizes for the referenced studies were small and that each study had a different follow-up period, which could conflate findings. Thus, more research is needed to examine mortality rate of ALWH using nationally representative data.

Though this systematic review was conducted carefully, it is not without limitations. First, most of the studies included in this review had small sample sizes and utilized a wide range of study designs and measures, precluding us from drawing unequivocal conclusions about ALWHs’ transition outcomes. Second, we limited our sample to studies that were published in peer-reviewed journals. Therefore, it is possible that newer studies may have been missed. Third, we included only studies that were published in English. Thus, it is possible that relevant studies were not included in this review.

## Conclusions

This systematic review suggests that ALWHs have fair to good outcomes post-HCT. However, to more accurately evaluate and compare ALWHs’ transition outcomes across studies and populations, standardized reporting is necessary. Moreover, the absence of adequately powered clinical trials limits our ability to draw conclusions about the effectiveness of various interventions that have been developed to transition ALWH to adult care. For this reason, controlled trials are needed. As evidenced by the small sample size in the current review, more research is needed to accurately characterize ALWHs’ outcomes post-HCT, particularly in high-incidence settings. In a time when ALWH around the world are reaching adolescents and adulthood, information regarding the outcomes of those who have transitioned could inform the development of future interventions. Through ensuring methodological rigor in transitionary HIV care, researchers will be better equipped to evaluate and compare ALWH transition outcomes across studies, and to increase program uptake, replicability, dissemination, and implementation.

## Supplementary information

**Additional file 1.** Assessment of the quality of manuscripts reviewed. This document provides a summary of the criteria used to assess the quality of each manuscript, along with the score of each manuscript.

**Additional file 2.** PRISM 2009 Checklist. In this document, we include a checklist demonstrating our compliance with PRISMA guidelines.

## Data Availability

Studies reviewed in the current study have been published and are widely available. A review protocol is not available for this study.

## Abbreviations

HIV: Human immunodeficiency virus
AIDS: Acquired Immune deficiency syndrome
ALWH: Adolescents living with HIV
HCT: Healthcare transition
VL: Viral load
CD4 +: Cluster of differentiation 4.
PHIV: Adolescents with perinatal HIV infection
BHIV: Adolescents with behavioral HIV infection
ART: Anti-retroviral therapy
LTFU: Lost-to-follow-up
